# On the Ophthalmoscope

**Published:** 1882-02

**Authors:** 


THE OPHTHALMOSCOPE; ITS THEORY AND PRACTICAL USES. By *C. H. Vilas, M. A., M. D.*, Prof. of Diseases of the Eye and Ear, in the Hahnemann Medical College, etc., etc. Pp. 150, 12mo. Chicago: Duncan Brothers, 1882.

Those who wish to learn what can be known through the use of the Opthalmoscope, may, by possessing this little work, and following the well known rules it contains, become familiar therewith. We can cordially commend Dr. Vilas' work, particularly to those who do not make frequent use of the Opthalmoscope. G. H. C.

